# Effect of high-pressure D_2_ and H_2_ annealing on LFN properties in FD-SOI pTFET

**DOI:** 10.1038/s41598-022-22575-5

**Published:** 2022-11-02

**Authors:** Hyun-Jin Shin, Sunil Babu Eadi, Yeong-Jin An, Tae-Gyu Ryu, Do-woo Kim, Hi-Deok Lee, Hyuk-Min Kwon

**Affiliations:** 1 Department of Electronics Engineering, Chungnam National University, 99, Daehak-ro, Yuseong-gu, Daejeon, Republic of Korea; 2 Department of Semiconductor Design, Semiconductor Convergence Campus of Korea Polytechnic College, 41-12, Songwon-Gil, Kongdo-Eup, Anseong, Kyunggi-Do Republic of Korea; 3 Department of Semiconductor Processing Equipment, Semiconductor Convergence Campus of Korea Polytechnic College, 41-12, Songwon-Gil, Kongdo-Eup, Anseong, Kyunggi-Do Republic of Korea

**Keywords:** Engineering, Electrical and electronic engineering, Physics, Applied physics, Electronics, photonics and device physics

## Abstract

Tunneling field-effect transistors (TFETs) are a promising candidate for the next generation of low-power devices, but their performance is very sensitive to traps near the tunneling junction. This study investigated the effects of high-pressure deuterium (D_2_) annealing and hydrogen (H_2_) annealing on the electrical performance and low-frequency noise (LFN) of a fully depleted silicon-on-insulator p-type TFET. Without high-pressure annealing, the typical noise power spectral density exhibited two Lorentzian spectra that were affected by fast and slow trap sites. With high-pressure annealing, the interface trap density related to fast trap sites was reduced. The passivation of traps near the tunneling junction indicates that high-pressure H_2_ and D_2_ annealing improves the electrical performance and LFN properties, and it may become a significant and necessary step for realizing integrated TFET technology in the future.

## Introduction

Tunneling field-effect transistors (TFETs) have attracted significant attention as the next generation of low-power devices because they can realize a very low off-current and subthreshold swing (SS) of less than 60 mV dec^−1^^1–3^. Because a very low SS is difficult to achieve in practice without the use of a special substrate or structure^1–3^, TFET operation is extremely sensitive to the materials used, geometry, and traps near the source/channel (tunneling) junction. Many studies have been conducted to improve the electrical efficiency of TFETs by modifying the device structure, introducing new substrate materials, or improving fabrication technology^1–3^. However, other important factors such as the 1/*f* noise and random telegraph signal noise (RTN) have received less attention despite being a significant limiting factor in analog and digital circuits. The effects of trap sites within the gate oxide and the current induced via fluctuation have emerged as critical concerns as devices continue to be scaled down^4,5^. The gate and potential barrier between the channel and source control the band-to-band tunneling mechanism in TFETs while drift–diffusion is used in conventional metal–oxide–semiconductor field-effect transistors (MOSFETs). The low-frequency noise (LFN) properties of TFETs and MOSFETs are generally dominated by the gate dielectric. For TFETs, the trapping and de-trapping characteristics of trap sites away from the tunnel junction may have a weak effect on the drain current (*I*_*DS*_) fluctuation. A few active traps around the tunnel junction of a TFET can influence the junction electric field and result in current fluctuations. Since the tunneling junction in TFET has a significant impact on electrical performance, the major LNF mechanism in nTFETs has been explained by carrier number fluctuations^6^. However, as the LNF properties in pTFETs have been still unclear, studies of the improvement on the LFN properties are still merit.

RTN also causes TFETs to have a high amplitude and significant device-to-device variability. Recently, deuterium (D_2_) and hydrogen (H_2_) annealing has been used to improve the reliability and LFN properties of silicon devices, including nanowire FETs^7,8^. Several studies have reported that high-pressure annealing improves the electrical performance with the benefit of a short annealing time because of the high concentration of D_2_ or H_2_ gas within a particular space^9,10^. The binding energy of the Si–D bond is known to have a higher kinetic isotope effect than that of the Si–H bond. In other words, the Si–D bond provides an energy-relaxation pathway that makes it more difficult to detach^11^. However, studies on p-type TFETs (pTFETs) have been limited compared with those on general n-type TFETs (nTFETs). Moreover, the effects of high-pressure H_2_ and D_2_ annealing on the LFN and RTN characteristics of pTFETs have not been reported.

In this study, we investigated the effects of high-pressure D_2_ and H_2_ annealing on the LFN properties of a fully depleted silicon-on-insulator (FD-SOI) pTFET. Multilevel RTN due to one fast trap site and one slow trap site was observed in the case without high-pressure annealing. High-pressure deuterium annealing (HPDA) had a curing effect on both fast and slow trap sites for a wide range of gate oxide depths. The interface trap density related to the fast trap sites was extracted by using the charge pumping method. Furthermore, we extracted the slow trap sites induced by fluctuation in the pTFET operation region. Our findings indicate that high-pressure annealing may be a significant and essential step toward improving the electrical performance and LFN properties of pTFETs.

## Methods

**Figure 1:**
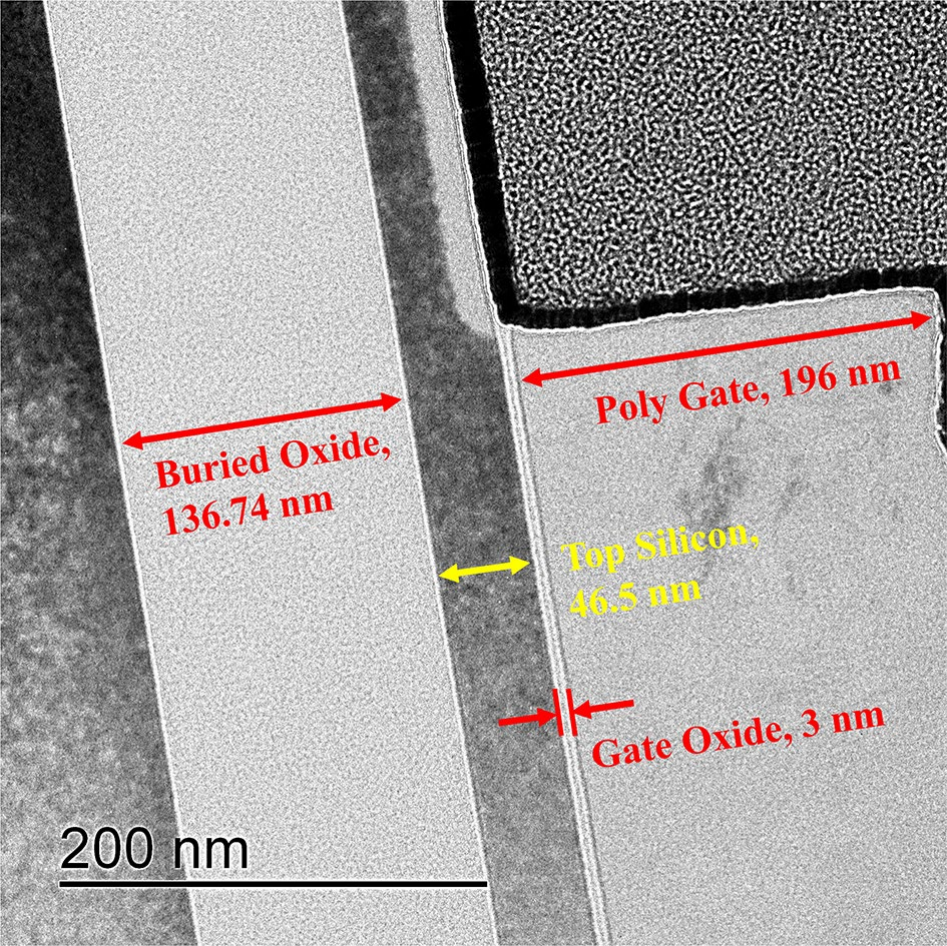
TEM image of the cross-section of an FD-SOI TFET.

TFETs were fabricated by using the FD-SOI technology. The top silicon layer was 46.5 nm thick with a doping concentration of about 10^16^ cm^−3^. The gate oxide layer was 3 nm thick and consisted of SiO_2_. n + poly-silicon (Si) was grown by low-pressure chemical vapor deposition. After the poly-Si gate electrode was patterned, arsenic implantation was applied from the source, and the drain was doped with BF_2_ implantation. The doping concentration was about 3 × 10^20^ cm^−3^ for both the source and drain. The pTFET had a width of 50 µm and length of 0.25 µm. A Cs-corrected scanning transmission electron microscopy image of the FD-SOI pTFET is shown in Fig. 1. After metal patterning, rapid thermal annealing for activation was performed for 10 s at 950 °C. To improve the LFN characteristics, post-metal annealing was performed for 30 min at 400 °C and 10 atm using D_2_ gas (6% D_2_ and 94% N_2_) or H_2_ gas (6% H_2_ and 94% N_2_).

**Figure 2:**
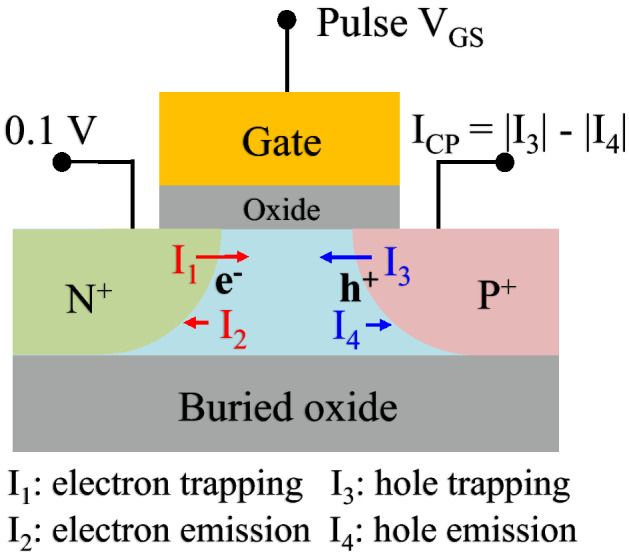
Schematic of the charge pumping method with the FD-SOI pTFET. When a pulse is applied to the pTFET gate, the charge pumping current is measured in the p^+^ region with the body contact. The reverse voltage at the n^+^ region is set to 100 mV, which is sufficient to eliminate the geometric component.

An Agilent 4156C semiconductor parameter analyzer was used to evaluate the electrical characteristics. A noise measurement system was used to characterize the LFN^12^. To separate the variability of the drain current, the typical noise power spectral density (PSD) was averaged 15 times. The normalized drain current noise (*S*_*ID*_*/I*_*DS*_^2^) was measured at |*V*_*DS*_|= 0.3 V, and *V*_*GS*_ was a constant at |*I*_*DS*_|= 1 µA. The RTN was measured for up to 2 s at the observed time domain and voltage at a specific frequency under the same conditions. The RTN was not averaged because it occurs during the short capture and emission events induced by a channel carrier. The charge pumping method for no-body contact was previously studied for a floating-body device^4,13^ and the three-dimensional interface of a fin structure^14^. Despite the no-body contact, the charge pumping method for TFETs is similar to the conventional charge pumping method for CMOSFETs. A charge pumping current could be measured in the p^+^ region similar to the body contact when a pulse was applied to the TFET gate, as shown in Fig. 2. A potential of 100 mV was applied to the n^+^ region, which was sufficient to eliminate the geometric component^15^. The interface trap density (*N*_*it*_) was extracted by using a fixed-amplitude charge pumping measurement method. A 1-MHz square waveform was applied to the gate terminator by an 81104A pulse generator (Agilent), and the charge pumping current was simultaneously measured by using the Agilent 4156C semiconductor parameter analyzer. The periodic trapezoidal pulses had a rising/falling time of 50 ns, amplitude of 1.3 V, base level of − 1.8 V, and duty cycle of 50%.

## Results and discussion

**Figure 3:**
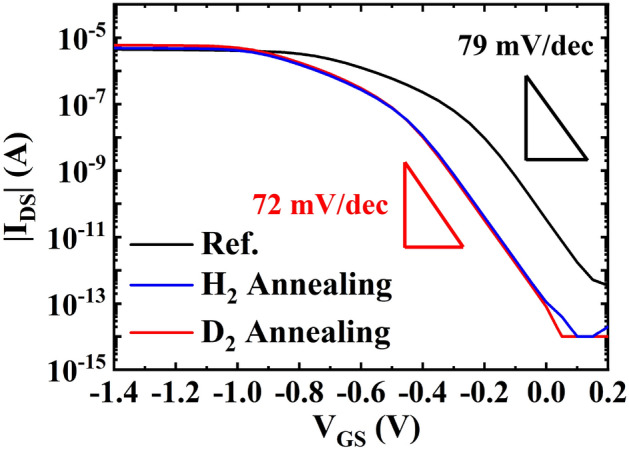
Subthreshold swing and electrical performance of the FD-SOI pTFET: without annealing (black), with HPHA (blue), and with HPDA (red).

Figure 3 compares the electrical performances of the FD-SOI pTFET without annealing (black), with high-pressure hydrogen annealing (HPHA, blue), and with HPDA (red). The SS of the pTFET was 79 mV dec^−1^ without annealing and 72 mV dec^−1^ with HPDA. The on-current also increased by ~ 33% from 4.44 µA without annealing to 5.92 µA with HPDA, and Vtcc at 1 nA shifted by − 200 mV. This demonstrates that HPDA resulted in effective passivation.

**Figure 4:**
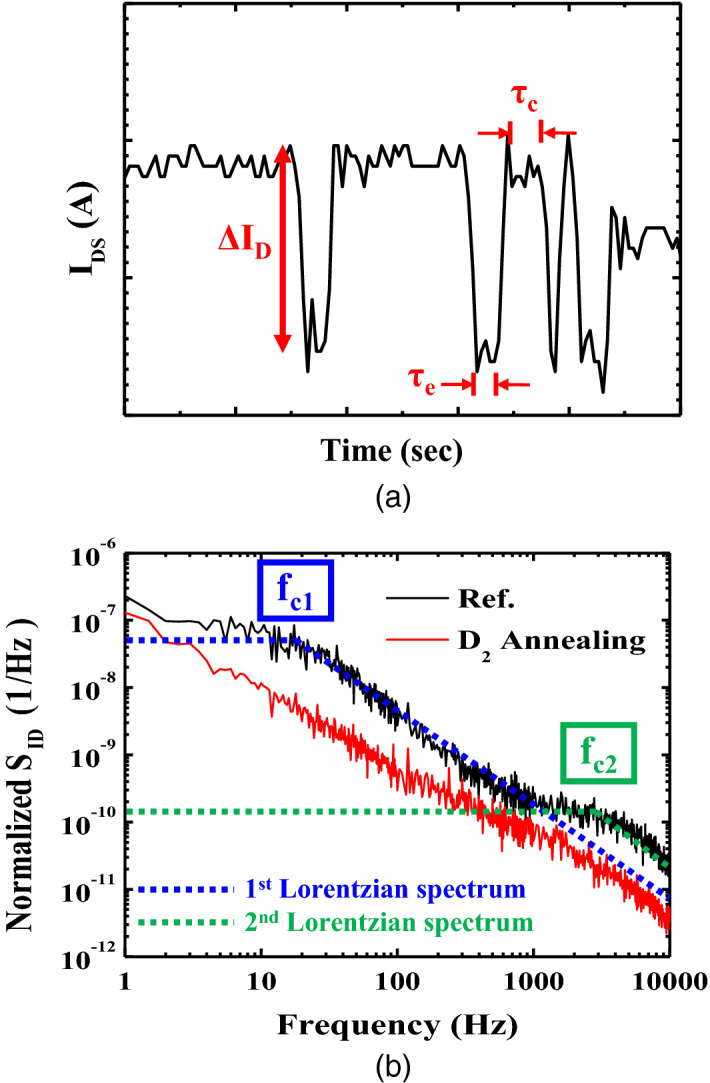
(**a**) Example of two discrete level fluctuations of drain current in the time domain induced by a single trap site. (**b**) Typical noise power spectral densities of the FD-SOI pTFET without annealing (black) and with HPDA (red). Two *f*_*c*_ without annealing can be observed with the superposition of different Lorentzian spectra, and the PSD is steeper with the HPDA than without the HPDA.

For a single trap, RTN was observed at two discrete levels in the time domain, as shown in Fig. 4a. RTN exhibited a high or low state in the time domain denoted by *τ*_*c*_ and *τ*_*e*_, respectively. If the dominant trap sites within a gate oxide have different levels, the current can fluctuate between two or more states, similar to an RTN waveform, because of random trapping and/or de-trapping of carriers within trap centers. The noise PSD of the current fluctuation can be calculated from the time domain data as follows^16^:

1 $$\frac{{S}_{ID}}{{I}_{D}^{2}}=\left(\frac{4{\tau }_{r}^{2}}{{\tau }_{t}}\right){\left(\frac{\Delta {I}_{D}}{{I}_{D}}\right)}^{2}\left[\frac{1}{1+{\left(2\pi f{\tau }_{r}\right)}^{2}}\right] $$

2 $${f}_{c}= \frac{1}{2\pi }\left(\frac{1}{{\tau }_{c}}+\frac{1}{{\tau }_{e}}\right)$$

where *τ*_*c*_ is the capture-time constant (i.e., time until an electron is captured within a trap site) and *τ*_*e*_ is the emission time constant (i.e., time until the electron is emitted from the trap site). These can be used to obtain *τ*_*r*_ = *τ*_*c*_*τ*_*e*_/(*τ*_*c*_ + *τ*_*e*_) and *τ*_*t*_ = *τ*_*c*_ + *τ*_*e*_. *f* is the frequency, *f*_*c*_ is the plateau region or corner frequency of the Lorentzian spectrum where the noise level is independent of frequency, and *∆I*_*DS*_ is the amplitude of the current induced by fluctuation.

As shown in Fig. 4b, the PSD for RTN at two discrete levels has Lorentzian spectra with a corner frequency (blue or green dash lines). A longer *τ*_*c*_ is required as the trap moves further away from the channel, and *f*_*c*_ corresponds to the location information of trap sites by (2)^16^. Interestingly, several *f*_*c*_ can be observed with the superposition of different Lorentzian spectra when multilevel RTN is induced from a number of trap sites. As shown in Fig. 4b, the PSD without high-pressure annealing (black) had two Lorentzian spectra. The first spectrum was caused by a slow trap site near 20 Hz (*f*_*C1*_, blue), and the second was caused by a fast trap site near 3000 Hz (*f*_*C2*_, green). This means that the two trap sites were at different depths within the gate oxide near the source/channel junction. In contrast, Fig. 4b shows that the PSD with HPDA (red) exhibited only a flicker noise characteristic because the dominant trap sites within the gate oxide were passivated, which resulted in a uniform spatial distribution of the gate oxide traps.

**Figure 5:**
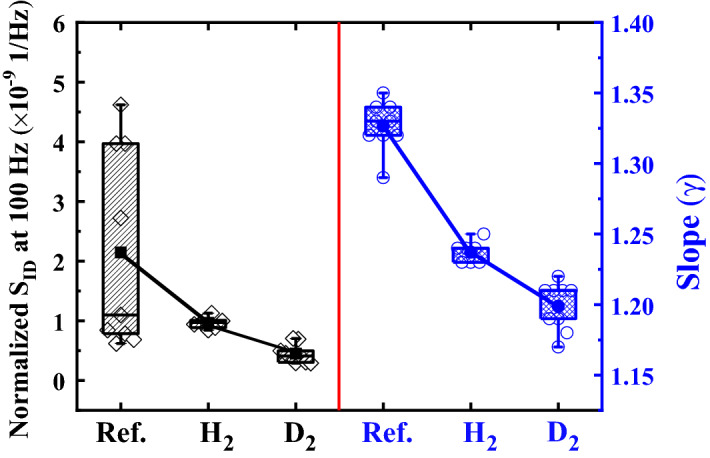
Normalized drain current noise (*S*_*ID*_*/I*_*DS*_^*2*^) and frequency exponent (γ) as a function of the annealing conditions.

Figure 5 shows that the normalized *S*_*ID*_*/I*_*DS*_^2^ at 100 Hz was 2.15 × 10^−9^ Hz^−1^ without annealing, 9.53 × 10^−10^ Hz^−1^ with HPHA, and 4.49 × 10^−10^ Hz^−1^ with HPDA. Thus, HPDA reduced the normalized *S*_*ID*_*/I*_*DS*_^2^ by ~ 79% compared to without annealing. The frequency exponent (γ) also decreased with high-pressure annealing, which means that the trap sites within the gate oxide were nearly uniformly distributed in terms of energy and depth^17^. γ was 1.327 without annealing, 1.237 with HPHA, and 1.199 with HPDA. These γ values are in the same range as those obtained for silicon-based devices such as MOSFETs^17^.

**Figure 6:**
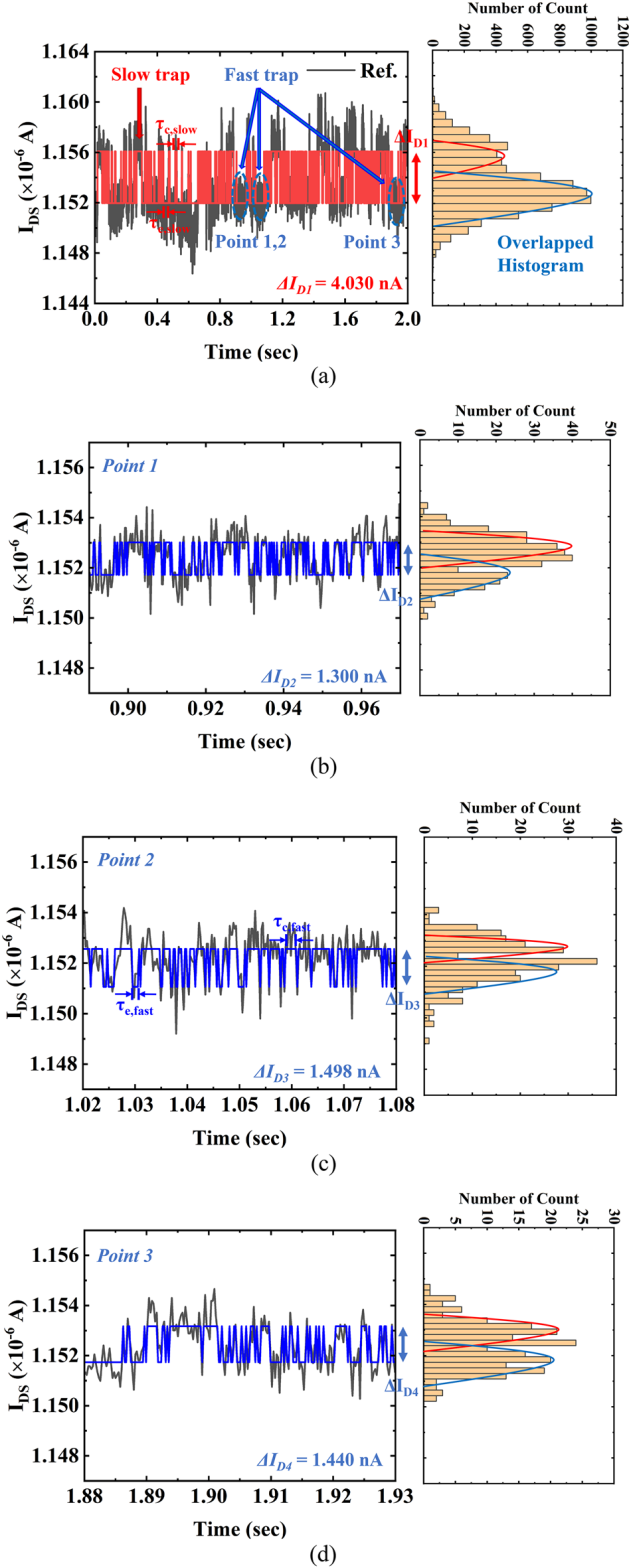
Time domain behavior of the drain current (*I*_*DS*_) RTN and corresponding histogram of *I*_*DS*_ for the FD-SOI pTFET without HPDA (the corresponding histogram of *I*_*DS*_ is also illustrated). (**a**) Multilevel RTN is induced by a slow trap site and fast trap site. (**b**) Point 1: Enlarged view of the fast trap site from 0.89 to 0.97 s. (**c**) Point 2: Enlarged view of the fast trap site from 1.02 to 1.08 s. (**d**) Point 3: Enlarged view of the fast trap site from 1.88 to 1.93 s.

**Figure 7:**
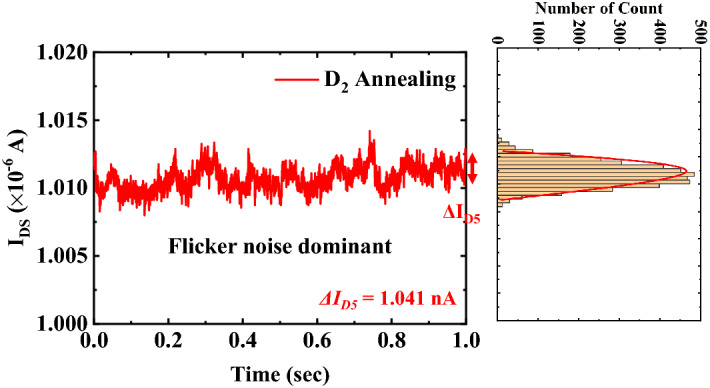
Time domain behavior of the drain current (*I*_*DS*_) RTN and corresponding histogram of *I*_*DS*_ for the FD-SOI pTFET with HPDA (the corresponding histogram of *I*_*DS*_ is also illustrated).

Figures 6 and 7 show the measured time domain drain current (*I*_*DS*_)-RTN and the corresponding histograms for the FD-SOI pTFET without and with HPDA. The randomly observed *I*_*DS*_-RTN was statistically analyzed to extract its distribution from the histogram^18,19^. The IDS-RTN measured for the pTFETs without and with HPDA was obtained by decoupling individual current levels using the change point detection method. Figure 6a shows the multilevel RTN due to one fast trap site and one slow trap site, along with an enlarged view of the fast trap site. The multilevel RTN indicated the non-uniformity of the traps. The multilevel RTN can also be identified in the enlarged views of the fast trap site, as shown in Fig. 6b–d. In general, bulk traps (i.e., slow traps) that are relatively deep require a long period for *τ*_*c*_ and *τ*_*e*_, whereas shallow interface traps (i.e., fast traps) require a short *τ*_*c*_ and *τ*_*e*_. The trap depth is connected to the RTN amplitude (*∆I*_*D*_*/I*_*D*_), and a larger RTN amplitude indicates a further distance from the existing trap in the gate oxide.

The variations in the drain current induced by slow and fast trap sites were *∆I*_*D1*_ = 4.030 nA, *∆I*_*D2*_ = 1.300 nA, *∆I*_*D3*_ = 1.498 nA, and *∆I*_*D4*_ = 1.440 nA. Figure 7 shows that the flicker noise was dominant after HPDA, which means that the typical characteristic of the RTN was no longer observed. This is consistent with the PSD with HPDA, as shown in Fig. 7. The amplitude was *∆I*_*D5*_ = 1.041 nA after HPDA, which shows that the *I*_*DS*_ amplitude was reduced compared with the case without annealing. This indicates that HPDA had a curing effect on both fast and slow trap sites for a wide range of gate oxide depths.

**Figure 8:**
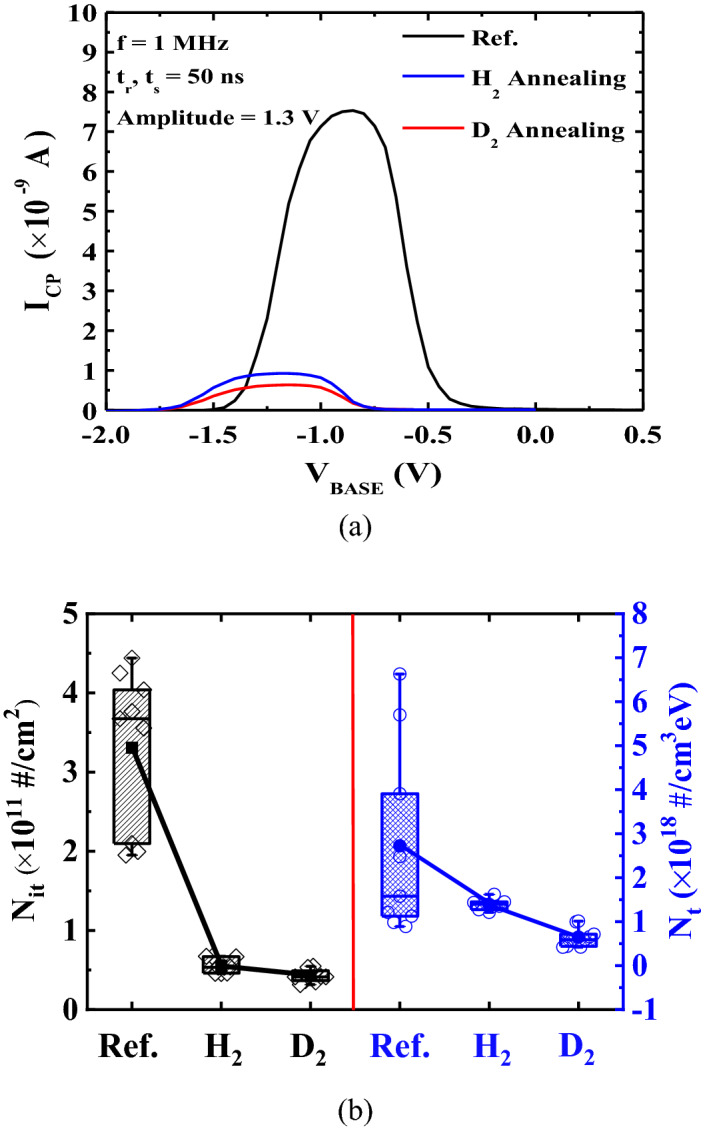
(**a**) Charge pumping current as a function of the gate pulse base voltage without and with HPHA and HPDA. The relation between the interface trap density and fast trap sites was verified by using the charge pumping method in pTFET. (**b**) Comparison of the interface trap density (*N*_*it*_) extracted by the charge pumping method and trap density extracted by the unified model as a function of the annealing conditions. *N*_*it*_ and *N*_*t*_ are related to the fast and slow trap sites, respectively, and indicate passivation via HPDA and HPHA.

To verify the interface trap density related to the fast trap sites, *N*_*it*_ was extracted by using the charge pumping method for no-body contact^13^. *N*_*it*_ can be calculated from *I*_*CP*_ in Fig. 8a as follows^13^:

3 $${N}_{it}= \frac{{I}_{cp, max}}{Aq{f}_{p}}$$

where *I*_*CP*_ is the charge pumping current, *A* is the gate area, *q* is the unit charge, and *f*_*p*_ is the pulse frequency. Figure 8b shows that the *N*_*it*_ values were 3.307 × 10^11^ cm^−2^ without annealing, 5.559 × 10^10^ cm^−2^ with HPHA, and 4.286 × 10^10^ cm^−2^ with HPDA. This means that *N*_*it*_ related to the fast trap sites was reduced because of passivation by HPDA and HPHA. The slow trap sites were attributed to the capture and emission of channel carriers by the trap sites in the gate oxide, which caused a large RTN amplitude as mentioned previously. The trap sites near the tunneling junction contributed to the LFN. Thus, pTFET design must consider the influence of the tunneling junction characteristics as well as channel transportation, and its impact on device design and circuit performance may need to be quantified. The small-signal model proposed by Wan et al.^20^ was used to calculate the total PSD of the pTFET, in which a tunneling diode and MOSFET are connected in series. The accuracy of the LFN model for the FD-SOI TFET was verified by Yaron and Frohman-Bentchkowsky^21^, and it can be calculated as follows:

4 $$\frac{{S}_{ID}}{{I}_{D}^{2}}={\left(\frac{1}{1+\kappa }\right)}^{2}\left\{ \left(\frac{\beta {\kappa }^{2}}{A}\right){\left(\frac{1}{N}+\alpha \mu \right)}^{2}\frac{1}{f}+\left(\frac{4{\tau }_{r}^{2}}{{\tau }_{t}}\right){\left(\frac{\Delta {I}_{D}}{{I}_{D}}\right)}^{2}\left[\frac{1}{1+{(2\pi f{\tau }_{r})}^{2}}\right]\right\}$$

5 $$\beta =kT\lambda {N}_{t} $$

where *κ* = *R*_*c*_/*R*_*t*_, *R*_*t*_ is the tunneling junction resistor, *R*_*c*_ is the channel resistor, and the pre-factor [1/(1 + *κ*)](4*τ*_*r*_^2^/*τ*_*t*_) = 5 × 10^−5^ Hz^−1^ and pre-factor [*κ*/(1 + *κ*)]^2^(*β*/*A*)[1/*N* + *αµ*]^2^ = 9 × 10^−9^ are assumed constant^21^. *β* is proportional to the trap density but is independent of |*V*_*GS*_|; *N* is the carrier density in the channel and is assumed to be 10^12^ cm^−2^ for a p-poly-SiO^2^ system^22–24^. *α* is the scattering coefficient, and it was reported to be 10^5^ Vs C^−1^ for holes^25^. *µ* is the carrier defective mobility^22^, and it was assumed to be 300 cm^2^ Vs^−1^. However, it is so small that it does not affect the final value in the calculation with (4). In other words, *N*_*t*_ can be obtained from (5), where *k* is the Boltzmann constant, *T* is the temperature, and *λ* is the tunneling attenuation length (≈ 0.1 nm in SiO_2_)^26^.

As shown in Fig. 8b, *N*_*t*_ from (5) was 2.72 × 10^18^ eV^−1^ cm^−3^ without annealing, 1.35 × 10^18^ eV^−1^ cm^−3^ with HPHA, and 6.55 × 10^17^ eV^−1^ cm^−3^ with HPDA. This means that *N*_*t*_ related to the slow trap sites was reduced because of passivation by HPDA and HPHA. Therefore, the results show that HPHA and HPDA are potentially significant and essential for future integrated TFET technology because they reduce typical noise characteristics such as the RTN and LFN as well as the thermal budget.

## Conclusion

This study evaluated the effects of HPDA and HPHA on the LFN properties of an FD-SOI pTFET. HPDA was found to improve the electrical performance and LFN properties. The PSD without high-pressure annealing had two Lorentzian spectra while the PSD with HPDA had a steeper slope. The multilevel RTN without high-pressure annealing was caused by one fast trap site and one slow trap site. The Nit related to the fast trap sites and Nt related to the slow trap sites were reduced by passivation via HPDA and HPHA. These results indicate that HPHA and HPDA are potentially significant and essential for future integrated TFET technology.

## Data Availability

The datasets generated during and/or analyzed during the current study are available from the corresponding author after reasonable request.
